# Fast Image Search with Locality-Sensitive Hashing and Homogeneous Kernels Map

**DOI:** 10.1155/2015/350676

**Published:** 2015-03-29

**Authors:** Jun-yi Li, Jian-hua Li

**Affiliations:** ^1^School of Electronic Information and Electrical Engineering, Shanghai Jiao Tong University, Shanghai 200240, China; ^2^Electrical and Computer Engineering Department, National University of Singapore, Singapore 119077

## Abstract

Fast image search with efficient additive kernels and kernel locality-sensitive hashing has been proposed. As to hold the kernel functions, recent work has probed methods to create locality-sensitive hashing, which guarantee our approach's linear time; however existing methods still do not solve the problem of locality-sensitive hashing (LSH) algorithm and indirectly sacrifice the loss in accuracy of search results in order to allow fast queries. To improve the search accuracy, we show how to apply explicit feature maps into the homogeneous kernels, which help in feature transformation and combine it with kernel locality-sensitive hashing. We prove our method on several large datasets and illustrate that it improves the accuracy relative to commonly used methods and make the task of object classification and, content-based retrieval more fast and accurate.

## 1. Introduction

In Web 2.0 applications era, we are experiencing the growth of information and confronted with the large amounts of user-based content from internet. As each one can publish and upload their information to the internet, it is urgent for us to handle the information brought by these people from internet. In order to organize and be close to these vision data from Internet, it has caused considerable concern of people. Therefore, the task of fast search and index for large video or image databases is very important and urgent for multimedia information retrieval such as vision search especially now the big data in some certain domains such as travel photo data from the website and social network image data or other image archives.

With the growth of vision data, we focus on two important aspects of problem including nearest neighbor search and similarity metric learning. For metric learning, many of the researchers have proposed some algorithms such as Information-Theoretic metric learning [1]. As for nearest neighbors search, the most common situation and task for us is to locate the most similar image from an image database. Among all the methods, given the similarity of example and query item, the most common method is to find all the vision data among the vision database and then sort them. However time complexity of this algorithm is too large and also impractical. When we handle image or video data, especially, this complexity will not be calculated, because it is very difficult for us to compute the distance of two items in higher dimensional space and also vision datum is sparse, so we cannot complete it by limited time.

Many researchers believe that linear scanning can solve this problem; although we believe it is a common approach and not suitable for computing in large-scale datasets, it promotes the development of ANN. LSH was used in ANN algorithms. To get fast query response for high-dimensional space input vectors [1–5], when using LSH, we will sacrifice the accuracy. To assure a high probability of collision for similar objects, randomized hash function must be computed; this is also referred to in many notable locality-sensitive hashing algorithms [6, 7].

Although, in object similarity search task, the LSH has played an important role, some other issues and problems have been neglected. In image retrieval, recognition, and search tasks, we find that they are very common:

- in the sample feature space, traditionally LSH approaches can only let us get a relatively high collision probability for items nearby. As a lot of vision datasets contained much rich information, we can find that the category tags are attached to YouTube and Flickr data and the class labels are attached to Caltech-101 images. However the low-level and high-level of vision samples have great gap, which means that the gap low-level features and high-level semantic information exist. To solve this problem, we intend to utilize the side additional information for constructing hash table;
- As to manipulate nonlinear data which is linear inseparable, we commonly use kernel method in vision task because of its popularity. For instance, in vision model, objects are often modeled as BOF and kernel trick is an important approach in classifying these data from low-dimension space to high-dimension space. However, how to create hash table in kernel spaces is a tough problem for us.

To verify our idea, we did several experiments in object search task. For example, we show our results on the Caltech-101 [8] dataset and demonstrate that our approach is superior to the existing hashing methods as our proposed algorithm.

In order to test our algorithm performance on dataset, we design some experiments on certain visual task such as Caltech-101 [8] and demonstrate that the performance of algorithm in our paper is beyond the traditional LSH approaches on the dataset, as hash functions can be calculated beyond many kernels. Arbitrary kernel in ANN is suitable in our scheme; actually we can find that a lot of similarity hashing functions can be accessed in the task of vision search tasks based on content retrieval.

## 2. Homogeneous Kernel

In our paper, we mainly focus on some similar kernels like intersection, Jensen-Shannon, Hellinger's, and *χ* ^2^ kernels. In the fields of machine learning and vision search, we often use these kernels as learning kernels. These kernels have two common attributes: being homogeneous and additive. The idea of kernel signature has been smoothly connected to these kernels in this section. Meanwhile we can use pure functions to represent these kernels. Also these attributes will be applied in Section 3 to obtain kernel feature maps. Through the kernel feature map, we can get their approximate expression.

*Homogeneous Kernels*. A kernel *k*_*l*_ : *ℝ*_0_^+^ × *ℝ*_0_^+^ → *ℝ* is *γ*-homogenous if

(1) $$\begin{array}{c} \forall m\geq 0:k_{l}\left(ma,mb\right)=m^{\gamma }k_{l}\left(a,b\right). \end{array}$$

When *γ* = 1, we believe that *k*_*l*_(*ma*, *mb*) is homogeneous. Let $m=1/\sqrt{ab}$; we can obtain a *γ*-homogeneous kernel and we can also write the formula as

(2) kla,b=m−γkl(ma,mb)=abγ/2klba,ab=abγ/2κlog⁡b−log⁡a.

Here the pure function

(3) $$\begin{array}{c} \kappa \left(\lambda \right)=k_{l}\left(e^{\lambda /2},e^{-\lambda /2}\right),\;\lambda \in R \end{array}$$

is called the kernel signature.

*Stationary Kernels*. A kernel *k*_*s*_ : *ℝ*_0_^+^ × *ℝ*_0_^+^ → *ℝ* is called stationary kernels if

(4) $$\begin{array}{c} \forall l\in R:k_{s}\left(l+a,l+b\right)=k_{s}\left(a,b\right). \end{array}$$

Let *l* = −(*a* + *b*)/2; the *k*_*s*_(*a*, *b*) is represented as

(5) ksa,b=ks(l+a,l+b)=ksb−a2,a−b2=κb−a,

(6) $$\begin{array}{c} \kappa \left(\lambda \right)=k_{s}\left(\frac{\lambda }{2},-\frac{\lambda }{2}\right),\;\lambda \in R. \end{array}$$

Here we call formula (6) kernel feature.

In the field of machine learning or computer vision, most of the homogeneous kernels are composed of the Jensen-Shannon, intersection, *χ* ^2^, and Hellinger's kernels. So we can also view them as additive kernels. In the next section, we will focus on these kernels and their kernel maps.  Table 1 shows the details [9].

*χ* ^2^ *Kernel*. We define *k*(*a*, *b*) = 2*ab*/(*a* + *b*) as the *χ* ^2^ kernel [10, 11]. Here the *χ* ^2^ distance is then defined as *D* ^2^(*a*, *b*) = *χ* ^2^(*a*, *b*).

*Jensen-Shannon (JS) Kernel*. We define *k*(*a*, *b*) = (*a*/2)log⁡_2_⁡(*a* + *b*)/*a* + (*b*/2)log_2_(*a* + *b*)/*b* as the JS kernel. Here the JS kernel distance *D* ^2^(*a*, *b*) can be obtained by *D* ^2^(*a*, *b*) = *KL*(*a*∣(*a* + *b*)/2) + *KL*(*b*∣(*a* + *b*)/2), where we import the concept of Kullback-Leibler divergence computed by *KL*(*a*∣*b*) = ∑_*l*=1_^*d*^ *a*_*l*_log_2_(*a*_*l*_/*b*_*l*_).

*Intersection Kernel*. We defined *k*(*a*, *b*) = min⁡{*a*, *b*} as the intersection kernel [12]. The distance metric *D* ^2^(*a*, *b*) = ‖*a* − *b*‖_1_ is *l* ^1^ distance between variants *a* and *b*.

*Hellinger's Kernel*. We defined $k(a,b)=\sqrt{ab}$ as the Hellinger's kernel and specified distance metric $D^{2}(a,b)={\left\|\sqrt{a}-\sqrt{b}\right\|}_{2}^{2}$ as Hellinger's distance between variants *a* and *b*. The function expression *κ*(*λ*) = 1 is the signature of the kernel, which is constant.

*γ*-*Homogeneous Parameters.* In previous research paper, we can see that the homogeneous kernels are used by parameters *γ* = 1 and *γ* = 2. When *γ* = 2, the kernel becomes *k*(*a*, *b*) = *ab*. Now, in our paper, we can derive the *γ*-homogeneous kernel by formula (2).

## 3. Homogeneous Kennel Map

When handling low-dimensional data which is inseparable, we should create kernel feature map *ψ*(*x*) for the kernel so that we can map our input data information in low-dimensional space to relatively high-dimensional (Hilbert) information space with 〈·, ·〉:

(7) $$\begin{array}{c} \forall a,b\in R^{D}:K\left(a,b\right)=\langle \psi \left(a\right),\psi \left(b\right)\rangle . \end{array}$$

In order to compute the feature maps and get approximate kernel feature maps expression for the homogeneous kernels, we should use Bochner's theorem by expanding the configuration of *γ*-homogeneous expression. Here we notice that if a homogeneous kernel is Positive Definite [13], its signature will also be Positive Definite expression. The assumption condition is suitable for a stationary kernel. So, depending on formulae (2) and Bochner's theorem (9), we can derive the *k*(*a*, *b*) and closed feature map.

We can compute the kernel density and feature map closed form [9] for most machine learning kernels.  Table 1 illustrates the results. Consider

(8) ka,b=abθ/2∫−∞+∞e−iwλκ(ω)dω, θ=log⁡ba=∫−∞+∞e−iwlog⁡aaθκω∗e−iwlog⁡bbθκωdω,

(9) $$\begin{array}{c} \psi_{w}\left(a\right)=e^{-iw\log a}\sqrt{a^{\gamma }\kappa \left(\omega \right)}. \end{array}$$

## 4. Kernelized Locality-Sensitive Hashing

To create and conduct the data association, we take the approach of Kernelized LSH [14] which is also a hash table-based algorithm. KLSH is proposed based on LSH algorithm, which is more efficient and accurate for query search and matching. When searching the input query, the KLSH approach can quickly locate the possible similar and nearest neighbor items in the hash table and match it. In addition, KLSH has another characteristic: traditional LSH methods can only find a part of hashes in the kernel space, while KLSH can locate all the possible hash tables in kernel space. Moreover KLSH has been applied in the vision search tasks by large scale datasets such as Tiny Image and other datasets [14].

Similar to LSH, constructing the hash functions for KLSH has been the key problem for us. That means if we intend to compute the collision probabilities of input query and the database points, we should compute the extent of similarity between them in the database as proposed by [15].

*KLSH Principle*. Any locality-sensitive hashing algorithm is based on the probability of distribution of hash function clusters. So we should compute the collision probability of a bundle of points, for example, *m* and *n*:

(10) $$\begin{array}{c} P_{r}\left(h\left(m\right)=h\left(n\right)\right)=\text{sim}\left(m,n\right). \end{array}$$

We can also view the problem as the issue of computing the similarity of objects between *m* and *n*. Here sim(*m*, *n*) in the algorithm is the measure function of calculating the similarity, while *h*(*m*) and *h*(*n*) are randomly selected from the hash function cluster *H*. The instinct beyond this is that we find the fact that *m* and *n* will collide in the same hash bucket. So those objects which are significantly similar will be more possible to be memorized in the hash table and this eventually results in confliction [1].

We can derive the similarity function expression according to the vector inner product:

(11) $$\begin{array}{c} \text{sim}\left(m,n\right)=m^{T}n. \end{array}$$

In [15, 16], the definition of LSH function has been extended from formula (10) as

(12) $$\begin{array}{c} h_{\vec{r}}\left(m\right)=\left\{\begin{array}{cc} 1, & \text{if}\;\;{\vec{r}}^{T}m\geq 0,\\ 0, & \text{else}. \end{array}\right. \end{array}$$

Here we create a random hyper plane vector $\vec{r}$. The distribution of $\vec{r}$ fit has a zero-mean multi-Gaussian *N*(0, Σ_*p*_) distribution. The dimensionality of $\vec{r}$ is the same with the input vector *m*. This demonstrates that the statistical characteristic of input vector is uniquely matched with each hash function. Meanwhile this verification has been detailedly reported in the LSH attribute in [17]. When we project on a point *m*, actually the sigh function we obtain in this process is a hash function and then we repeat it *k* times; a couple of hashes can be created. We can also call this couple of hashes hash bucket. The hash bucket can be formed as

(13) $$\begin{array}{c} g\left(m\right)=\langle h_{1}\left(m\right),\ldots ,h_{t}\left(m\right),\ldots {,h}_{k}\left(m\right)\rangle . \end{array}$$

From (13), we can see that, after repeating *k* times, we can get one column of hash bucket (14); then repeating *b* times, we can finally obtain the hash bucket *g*(*m*):

(14) $$\begin{array}{c} g_{j}\left(m\right)=\langle h_{1}\left(m\right),\ldots ,h_{t}\left(m\right),\ldots ,h_{k}\left(m\right)\rangle \;\left(1<j<b\right). \end{array}$$

When given the value of *b*, we can get all the the hash functions located in the bucket; we can see the following:

(15) $$\begin{array}{c} g\left(m\right)=\left\{\begin{array}{cccccc} h_{1,1\vec{r}}\left(m\right) & h_{2,1\vec{r}}\left(m\right) & \cdots & h_{s,1\vec{r}}\left(m\right) & \cdots & h_{t,1\vec{r}}\left(m\right)\\ \vdots & \vdots & \vdots & \vdots & \vdots & \vdots \\ h_{1,j\vec{r}}\left(p\right) & h_{2,j\vec{r}}\left(m\right) & \cdots & h_{s,j\vec{r}}\left(m\right) & \cdots & h_{t,j\vec{r}}\left(m\right)\\ \vdots & \vdots & \vdots & \vdots & \vdots & \vdots \\ h_{1,b\vec{r}}\left(m\right) & h_{2,b\vec{r}}\left(m\right) & \cdots & h_{s,b\vec{r}}\left(m\right) & \cdots & h_{t,b\vec{r}}\left(m\right) \end{array}\right\},\;\left(1<j<b;1<s<t\right). \end{array}$$

Due to the fact that we compute the similarity measure function in high-dimensional kernel space, the similarity function can also be extended and written as

(16) $$\begin{array}{c} \text{sim}\left(\left(m_{i},m_{j}\right)\right)=\kappa \left(m_{i},m_{j}\right)=\phi {\left(m_{i}\right)}^{T}\phi \left(m_{j}\right). \end{array}$$

In formula (16), we use kernel function *ϕ*(*m*) to construct *κ*(*m*_*i*_, *m*_*j*_) to complete the kernel mapping for the points of m_*i*_ and *m*_*j*_. And *ϕ*(*m*_*i*_)^*T*^ *ϕ*(*m*_*j*_) is a product of projection on hash function from the *ℜ* space. The problem is that nothing is known about the data while in kernel space to generate $\vec{r}$ from *N*(0, Σ_*p*_). Therefore, in order to construct the hash function, $\vec{r}$ needs to be created so that we can quickly compute the ${\vec{r}}^{T}\phi \left(m\right)$ function based on the kernel. Similar to normal ${\vec{r}}^{T}$, we could use only the kernel of *ϕ*(*m*) to approximately compute the function of ${\vec{r}}^{T}\phi (m)$. We should select a subset of database to construct $\vec{r}$. By the large number of central limit theory, if we intend to choose parts of database items from the whole database to form the dataset *S*, the sample of kernel data must be satisfied by the distribution with mean *μ* and variance Σ. The variable *z*_*a*_ can be written as

(17) $$\begin{array}{c} z_{a}=\frac{1}{k}\overset{}{\underset{i\in s}{\sum }}\phi \left(m_{i}\right). \end{array}$$

With the growth of variable *a*, the theory tells us that the vector ${\tilde{z}}_{a}=\sqrt{t}(z_{a}-\mu )$ has also been satisfied by the distribution of normal Gaussian.

We used the whitening transform to obtain $\vec{r}$:

(18) $$\begin{array}{c} \vec{r}=\Sigma^{-1/2}{\tilde{z}}_{a}. \end{array}$$

The LSH function has been yielded:

(19) $$\begin{array}{c} h\left(\phi \left(m\right)\right)=\left\{\begin{array}{cc} 1, & \text{if}\;\;\phi {\left(m\right)}^{T}\Sigma^{-1/2}{\tilde{z}}_{a}\geq 0,\\ 0, & \text{else}. \end{array}\right. \end{array}$$

As analyzed above, we use kernel function to represent the database data; then the statistical data like variance and mean are uncertain. If we intend to estimate and calculate *μ* and Σ, we could sample the data from the database by KPCA and eigen decomposition in [18] and we let Σ = *V*Λ*V* ^*T*^  and Σ^−1/2^ = *V*Λ^−1/2^ *V* ^*T*^; therefore we can obtain the hash function *h*(*ϕ*(*m*)):

(20) $$\begin{array}{c} h\left(\phi \left(m\right)\right)=\mathrm{sign}\left(\phi {\left(m\right)}^{T}V\Lambda^{-1/2}V^{T}{\tilde{z}}_{a}\right). \end{array}$$

From the above, we can see how to construct the hash function for the kernel matrix input vectors. In this case, we let the kernel matrix input be *K* = *UΩU* ^*T*^  by decomposing the *K* matrix. Here *Ω* and Λ have the same nonzero eigenvalue; it is also viewed as another form of kernel matrix input. From [18], we compute the projection

(21) $$\begin{array}{c} v_{t}^{T}\phi \left(m\right)=\overset{n}{\underset{i=1}{\sum }}\frac{1}{\sqrt{\theta_{t}}}u_{t}\left(i\right)\phi {\left(m_{i}\right)}^{T}\phi \left(m\right). \end{array}$$

Here *u*_*t*_ and *v*_*t*_ are, respectively, the *t*th eigenvector of the kernel matrix and its covariance matrix.

As mentioned before, we choose *n* data points from the database to form *ϕ*(*m*_*i*_); traversing all the *t* eigenvectors and conducting the computation yields

(22) hϕm=ϕmTVΛ−1/2VTz~a=∑t=1nθtvtTϕmTvtTz~a.

Substituting (21) into (22) yields

(23) ∑t=1nθt(∑i=1n1θtut(i)ϕmiTϕ(m))  ·∑i=1n1θtutiϕmiTz~a.

Simplifying (23) yields

(24) ∑i=1n∑j=1n(ϕmiTϕ(m))  ·ϕmjTz~a∑t=1n1θtutiutj.

Since $K_{ij}^{-1/2}=\sum_{k=1}^{n}\left(1/\sqrt{\theta_{k}}\right)u_{k}(i)u_{k}(j)$, we further simplify the (24) yields

(25) $$\begin{array}{c} h\left(\phi \left(m\right)\right)=\overset{n}{\underset{i=1}{\sum }}w\left(i\right)(\phi {\left(m_{i}\right)}^{T}\phi \left(m\right)), \end{array}$$

where $w(i)=\sum_{j=1}^{n}K_{ij}^{-1/2}\phi {\left(x_{j}\right)}^{T}{\tilde{z}}_{a}$.

Through the above derived formula *w*(*i*) we can obtain $\vec{r}=\sum_{i=1}^{n}w(i)\phi (x_{i})$ which obeys random Gaussian distribution, then we can substitute (17) into *w*(*i*):

(26) $$\begin{array}{c} w\left(i\right)=\frac{1}{a}\overset{n}{\underset{j=1}{\sum }}\;\underset{l\in s}{\sum }K_{ij}^{-1/2}K_{jl}. \end{array}$$

We neglect the term of $\sqrt{a}$, and finally the simplified *w*(*i*) yields (27). *e*_*s*_ represents the unit vector for *S*.

And therefore hash function for kernel input will finally be

(27) $$\begin{array}{c} w=K^{1/2}\cdot \frac{e_{s}}{k}, \end{array}$$

(28) $$\begin{array}{c} h\left(\phi \left(m\right)\right)=\mathrm{sign}\left(\overset{n}{\underset{i=1}{\sum }}w\left(i\right)\kappa \left(m{,m}_{i}\right)\right). \end{array}$$

*κ* is the kernel mapping matrix for points *m* and *m*_*i*_ in space. After several iterations, the hash function will form a hash bucket.

In order to get the suitable parameters in this process, we implement the query matching for several iterations. The detailed algorithm is illustrated finally in Algorithm 1. Consider

(29) gjm=h1ϕm,h2,jϕm,…,ht,jϕm,…,hk,jϕm,fffffffffffffffffffffffffffff1<l<t, 1<j<b.

## 5. Experimental Result

In the experiment, we proposed the homogenous kernel-hashing algorithm and verified the high efficiency on the dataset. In our scheme, homogenous kernel-KLSH method makes it possible to get the unknown feature embeddings. We use these features to conduct vision search task to locate the most similar items in the database, and the neighbors we find in the task will give their scores on the tags. The method proved to be more effective and accurate than the linear scan search.

**Figure 1:**
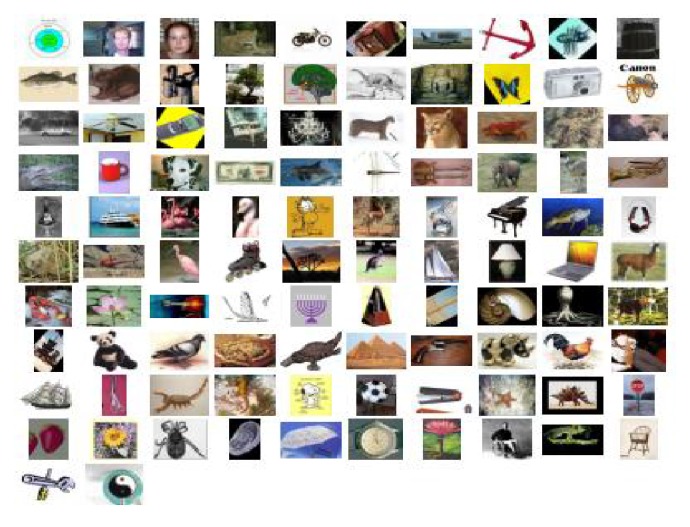
Datasets: Caltech-101 Example.

In this part, we design and verify our algorithm on the Caltech-101 dataset in Figure 1. Caltech-101 dataset is a benchmark on image recognition and classification, which has 101 categories objects and each category has about 100 images, so 10000 images totally. In recent years, many researchers have done useful research on this dataset such us proposing some important and useful image represent kernels [19]. Also there are many published papers that focused on this dataset, some of which are very valuable and significantly historic. For example, papers [20–22], respectively, state their contribution to the dataset. The author of [21] proposed the matching method for pyramid kernel of images histograms, while Berg [20] proposed and created the CORR kernel of image local feature using geometric blur for matching local image similarity.

In our paper, we apply our algorithm to complete the vision classification and similar search task. The platform of our benchmark is based on Intel 4 core 3.6 GHZ CPU and 16 GB of memory and 2 TB hard disk.

We used *χ* ^2^ kernel for *γ*-homogeneous kernel maps (*γ* = 1/2) and applied the nonlinear RBF-*χ* ^2^ kernel designed in [19, 23] to the SIFT-based local feature. Meanwhile we applied and learnt the homogenous kernel map beyond it. Compared with the nonlearnt kernel, our learnt kernel has been more accurate. And we use KNN classifier, respectively, for KLSH and linear scan to compute the accuracy of classification. We also compare it with CORR [24] and the result proves to be better than them, here we use 15 images per class for training task.

**Figure 2:**
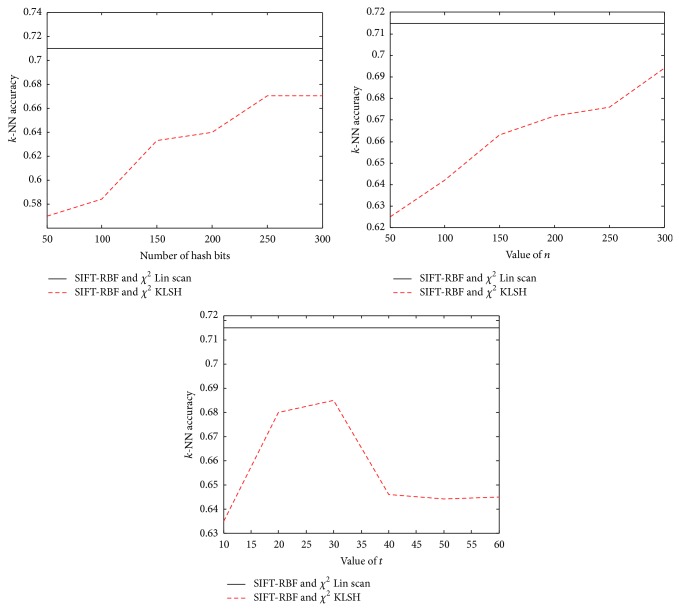
Hashing using a RBF-*χ* ^2^ kernel for SIFT based on homogenous kernels *χ* ^2^  (*γ* = 1/2). We choose *t* = 30, *n* = 300, and *b* = 300 in our experiment.

From Figure 2 we can see that the growth of parameters is closely related with accuracy. As is seen, the accuracy increased with the increase of *n*, while it has little relationship with the number of *t* and *b*. The value of (*n*, *t*, *b*) is chosen as *n* = 300, *b* = 300, *t* = 30 as the best parameters through a series of experiments.

We find that the combination of these parameters can result in better performance than the large-scale dataset. Meanwhile it can be seen that our approach with homogenous kernel map has higher accuracy than CORR-KLSH with metric learning [25].

**Figure 3:**
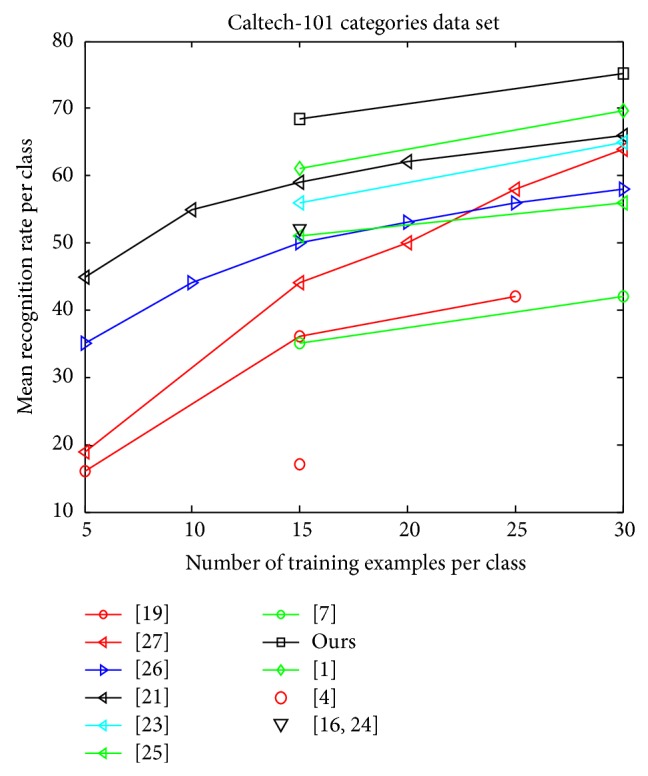
Comparison against existing techniques on the Caltech-101.

Figure 3 illustrates that our method is superior to other existing approaches [25–28] tested on this dataset. Comparing with other kernel classifiers, our classifier with RBF-*χ* ^2^ kernel for local features performs better. In Table 2 we can see that the result of ours has higher accuracy with *T* = 15 and *T* = 30 than other papers' results including better than [24] which obtains the result by 61% for *T* = 15 and 69.6% for *T* = 30. More clearly, it has improved the result by 16% several years ago.

**Figure 4:**
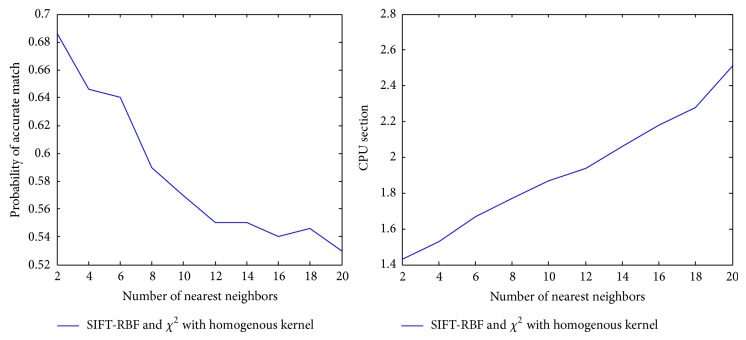
Classification beyond CPU load performance.

In order to find the best parameters in our experiment for NN search for our scheme, we should take into account the balance between performance and CPU time. Therefore here we conducted to analyze the performance and CPU time of different of *k*  (*k* = 2,3,…, 20) for NN search.  Figure 4 illustrates the accuracy and CPU time by each *k* in our dataset.

The author of [26] proposed the method by combining KPCA and normal LSH. That means computing hashing beyond the KPCA. However this method has apparent disadvantage because KPCA will bring on the loss of input information although it can reduce the dimensionality in the processing, while KLSH can solve this problem to assure the integrity of input information to compute the LSH. Therefore we found that our method has high accuracy and better performance than the algorithm in [26].

## 6. Conclusions

In our paper, we properly use the concept of homogeneous kernel maps to help us to solve the problem of approximation of those kernels, including those commonly used in machine learning such as *χ* ^2^, JS, Hellinger's, and intersection kernels. Combining with the KLSH scheme, it enables us to have access to any kernel function for hashing functions. Although our approach is inferior to linear scan search in time but it can guarantee that the search accuracy will not be affected. Moreover we do not need to consider the distribution of input data; to some extent, it can be applicable for many other databases as Flicker and Tiny Image. Experimental results demonstrate that it is superior to standard KLSH algorithm.

## Figures and Tables

**Algorithm 1:**
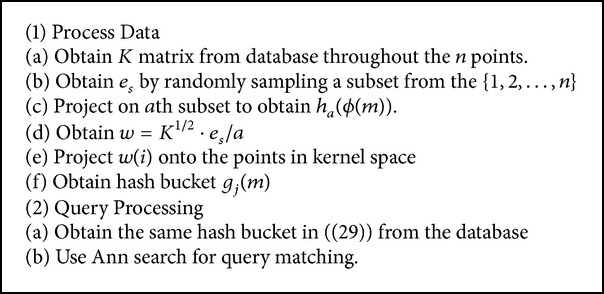
KLSH algorithm.

**Table 1:** Common kernels, signature, and their feature maps.

Kernel	*k*(*a*, *b*)	Signature *κ*(*θ*)	*κ*(*w*)	Feature *ψ*_*ω*_(*a*)
Hellinger's	$\sqrt{ab}$	1	*δ*(*ω*)	$\sqrt{a}$
*χ* ^2^	$\frac{2ab}{a+b}$	$\mathrm{sech}\left(\frac{\theta }{2}\right)$	sech⁡(*πω*)	$e^{iw\log a}\sqrt{a\mathrm{sech}\left(\pi \omega \right)}$
Intersection	min⁡{*a*, *b*}	*e* ^−|*θ*|/2^	$\frac{2}{\pi }\frac{1}{1+4\omega^{2}}$	$e^{iw\log a}\sqrt{\frac{2a}{\pi }\frac{1}{1+4\omega^{2}}}$
JS	$\frac{a}{2}\log_{2}\frac{a+b}{a}+\frac{b}{2}\log_{2}\frac{a+b}{b}$	$\frac{e^{\theta /2}}{2}\log_{2}\left(1+e^{-\theta }\right)+\frac{e^{-\theta /2}}{2}\log_{2}\left(1+e^{\theta }\right)$	$\frac{2}{\log4}\frac{\mathrm{sech}\left(\pi \omega \right)}{1+4\omega^{2}}$	$e^{iw\log a}\sqrt{\frac{2}{\log4}\frac{\mathrm{sech}\left(\pi \omega \right)}{1+4\omega^{2}}}$

**Table 2:** Accuracy of Caltech-101.

#train	Ours	[18]	[29]	[30]	[31]	[32]	[33]
15	68.5	59.05	56.4	52	51	49.52	44
30	75.2	66.23	64.6	N/A	56	58.23	63
